# Knowledge, Attitudes and Practices Regarding Cervical Cancer and Screening among Haitian Health Care Workers

**DOI:** 10.3390/ijerph111111541

**Published:** 2014-11-10

**Authors:** Leilah Zahedi, Emma Sizemore, Stuart Malcolm, Emily Grossniklaus, Oguchi Nwosu

**Affiliations:** 1 Department of Obstetrics and Gynecology, Emory University, 69 Jesse Hill Junior Drive South East, Atlanta, GA 30303, USA; 2 Rollins School of Public Health, Emory University, 1518 Clifton Rd. North East, Atlanta, GA 30322, USA; E-Mail: emma.sizemore@emory.edu; 3 School of Medicine, Emory University, 201 Dowman Drive, Atlanta, GA 30322, USA; E-Mails: stuart.malcolm@emory.edu (S.M.); emily.grossniklaus@emory.edu (E.G.); 4 Department of Family and Preventive Medicine, Emory University, 201 Dowman Drive, Atlanta, GA 30322, USA; E-Mail: oonwosu@emory.edu

**Keywords:** cervical cancer, Haiti, Central Plateau, KAP, VIA, cryotherapy, public health

## Abstract

It is estimated that Haiti has the highest incidence of cervical cancer in the Western Hemisphere. There are currently no sustainable and affordable cervical cancer screening programs in Haiti. The current status of screening services and knowledge of health care professionals was assessed through a Knowledge, Attitudes, and Practices survey on cervical cancer screening and prevention. It was distributed to Project Medishare for Haiti health care workers (*n* = 27) in the Central Plateau. The majority (22/27) of participants stated pre-cancerous cells could be detected through screening, however, only four had ever performed a pap smear. All of the participants felt a screening program should be started in their area. Our data establishes that knowledge is fairly lacking among healthcare workers and there is an opportunity to train them in simple, cost effective “screen-and-treat” programs that could have a great impact on the overall health of the population.

## 1. Introduction

In Haiti roughly 353 women die of cervical cancer each year, the second highest cancer-related mortality rate among Haitian women (7.1 per 100,000) [1]. In 2000, the International Agency on Research for Cancer estimated that Haiti has the highest incidence of cervical cancer in the Western Hemisphere at 93.8 per 100,000 [2]. It is likely that these estimates are significantly lower than the actual number of cervical cancer cases and deaths in Haiti, since there is a low level of awareness, limited access to screening services and no national cancer registry. Globally, cervical cancer is the third most common cancer in women; in 2008 there were an estimated 529,000 new cases [3]. Low-resource countries experience 85% of the global burden, and in regions such as Eastern Africa and South-Central Asia, cervical cancer is the most common cancer in women accounting for 13% of all female cancers [3]. If detected early cervical cancer is usually curable. However, the global mortality: incidence ratio is 52% [3]. In 2008, cervical cancer was responsible for 275,000 deaths, 88% of which occurred in low-resource regions of the world [3]. The majority of cervical cancer deaths occur in women who were never screened or treated, as well as those who had an early sexual debut, a history of multiple sexual partners, and a high number of live births [4].

Cervical cancer has several unique characteristics that make prevention through screening and the treatment of precancerous stages relatively straightforward [5]. The cause of virtually all cervical cancer cases is known to be the persistent infection with a restricted set of human papillomaviruses (HPVs) [6,7]. Cervical cancer also exhibits an identifiable precancerous condition and the time window from dysplasia to carcinoma is long (approximately 10 years on average). Several screening methods to detect precancer and cancer are available, and can be performed safely and inexpensively in an outpatient setting [5]. These methods include, but are not limited to, visual inspection with acetic acid (VIA), and careHPV. In addition to being used throughout many resource-limited countries around the world, these methods are also effective at treating precancerous findings, thus further decreasing the burden of disease [5]. Primary prevention through vaccination against the prevalent carcinogenic HPV types, HPV-16 and HPV-18, is also possible, but is significantly more expensive [8]. In order to best address the overwhelming disease burden in Haiti, the current status of screening services and health professional knowledge must be assessed.

“A Knowledge, Attitudes, and Practices (KAP)” survey is a representative study of a specific population that aims to collect data on what is known, believed and done in relation to a particular topic. KAP survey data are essential to help plan, implement and evaluate programs, and can identify knowledge gaps, cultural beliefs, or behavioral patterns that may facilitate or impede the success of a program.

## 2. Experimental Section

A KAP survey on cervical cancer screening and prevention was distributed to Project Medishare for Haiti staff, community health workers, Haitian physicians and nurses in the Central Plateau. It was provided to these health care workers at several Haitian medical clinics throughout the Central Plateau to assess baseline KAP about cervical cancer, screening and treatment. This project received approval to be carried out by the Emory University Institutional Review Board and the In-Country Coordinator for Project Medishare for Haiti, Marie Chery. Our project was modeled after a previous KAP study, conducted by Dr. Henry Blumberg, Dr. Jennifer Goedken, and others performed in Ethiopia [9].

Project Medishare for Haiti is a US-based Non-Governmental Organization that runs mobile and permanent clinics throughout the Central Plateau region, including Thomonde Clinic, Casse Maternity Ward and Clinic, and Marmont Clinic. The Central Plateau is a rural region of Haiti, home to several hundred thousand people. Communities exist as mostly small town centers around subsistence farms. While recent relief efforts have attempted to improve healthcare in the region, the problem of accessibility between and within communities remains a problem. Large paved roads exist between communities, but travel to rural destinations is impeded by lack of roads altogether. In many communities, the closest source of healthcare is more than a half-day’s walk away.

A cross sectional, observational study design was used to assess the knowledge, attitude and practices of cervical cancer, screening methodologies such as visible inspection of the cervix with acetic acid and Pap smear (see Supplementary Information). More specifically the survey addresses healthcare worker’s knowledge of risks, treatment and prognosis of cervical cancer, awareness of available screening methods, and their experience and attitudes to the different methodologies. The survey was conducted between June 22nd and 29th, 2013. All adult health care workers in the Central Plateau region were eligible to participate. Surveys were made available in French, Creole or English and were chosen to the participant’s preference. Data was anonymous and no personal identifiers were collected. Basic statistical analysis of survey results was performed in SAS. The survey in English is presented in the Supplementary Information.

KAP surveys were offered to nursing staff, midwives, community health workers and physicians at mobile and permanent clinic sites. All subjects gave their informed consent for inclusion before they participated in the study. The study was conducted in accordance with the Declaration of Helsinki. The protocol was approved by the Ethics Committee of the Emory University Institutional Review Board (IRB00067076). Time was allotted during breaks so as not to interfere with daily work commitments. The survey was also allowed to be taken home and completed. Surveys were collected on site or retrieved at local clinics from a safe and secure location. Those participants included were current health care workers (nurses, midwives, community health workers and physicians) and Project Medishare for Haiti staff in the Central Plateau region that were at least 18 years old. The study excluded any persons who were younger than 18 years old. No exclusion was made with respect to gender, race, ethnicity or social status.

## 3. Results and Discussion

### 3.1. Survey Results

#### 3.1.1. Knowledge

The study population included 27 participants in total. Table 1 presents the demographics of the study population. The mean age of the participants was 34 years old. 95% of participants were female and 100% identified themselves as Haitian. The majority (19/27) of participants spoke Creole, and 9/27 had only worked for 1–4 years. With regards to their knowledge about cervical cancer and prevention, 69.2% (18/26) stated they did not feel they had adequate knowledge. 100% of participants correctly stated that cervical cancer is one of the leading causes of death in women worldwide. Also, 52.2% (12/23) correctly stated that cervical cancer is preventable. When asked whether cervical cancer was curable, 45.5% (10/22) of the study sample correctly answered that question. When asked if it is possible to detect pre-cancerous cervical cancer cells, 81.5% (22/27) correctly stated that was true. 74.1% (20/27) of participants also recognized that cervical cancer is not most common among women in their 20 s. When asked whether cervical cancer can usually be found at an early stage because of the obvious symptoms such as bleeding and pain, 18.5% (5/23) correctly stated that was false. Two-thirds (18/27) correctly recognized that if cervical cancer is left untreated it is fatal. When participants were asked whether cervical cancer is caused by a virus that is spread sexually, 77.8% (21/24) correctly stated that was true. When asked whether there is a vaccine that can prevent cervical cancer, one-third (9/27) stated that was true. Almost all of participants (25/27) correctly recognized the purpose for screening is to detect pre-cancerous changes. When asked whether screening for cervical cancer should begin when a woman is in her twenties, 51.9% (18/27) stated this was false. When the women surveyed were asked whether they felt they were themselves at risk for cervical cancer, 71.4% (10/14) stated they did not think they are at risk. The risk factors for cervical cancer most often chosen by participants were: HIV infection (38.5%), smoking (53.9%), multiple partners (57.7%), and HPV infection (73.1%). Of those participants that stated they were not at risk for cervical cancer themselves, 80% (8/10) stated multiple partners and 90% (9/10) stated HPV infection were risk factors for cervical cancer. Of those who stated they were at risk for cervical cancer, 100% (4/4) stated poor hygiene and 75% (3/4) stated IUD use were risk factors for cervical cancer. Among those participants who earlier stated they felt they were knowledgeable about cervical cancer, 83.3% (5/6) correctly identified HPV infection and multiple partners as risk factors for cervical cancer. Among those participants that stated they were not knowledgeable or required additional education about cervical cancer, 66.7% correctly identified HPV infection and 44.4% correctly identified multiple partners as risk factors for cervical cancer. When asked what they thought were symptoms of advanced cervical cancer, 20% (5/25) identified abdominal pain, abnormal bleeding, foul-smelling discharge, and loss of appetite, 20% (5/25) identified abdominal pain, abnormal bleeding, and loss of appetite, and 16% (4/25) identified abnormal bleeding as symptoms. Finally, when asked whether they felt cervical cancer screening is an essential part of women’s health, 92.3% (24/26) strongly agreed.

**Table 1:** Demographics of Haitian Healthcare Workers.

Demographic	Total (N)
**Age**	
≤30	8
>31	11
**Gender**	
Female	19
Male	1
**Ethnic Group**	
Haitian	21
Other	-
**Primary Language**	
Creole	19
French	2
English	1
**Primary Job**	
Medical Student	1
Nurse	4
OBGYN Physician	1
Other	15
**Length of Time at Current Job**	
<1 year	5
1–4 years	9
5–10 years	4
>10 years	3

Note: Some participants did not complete all questions so numbers may not add to 27 due to missing answers.

#### 3.1.2. Attitudes

Among our participants, 52.4% stated they were very willing to do visual screenings on their patients. Of those participants who stated they needed more knowledge in order to perform screening, 72.2% (13/18) were willing to do visual screening. When asked whether a cervical cancer screening program should be started in their community, 100% of participants agreed or strongly agreed. Of those who had worked less than one year, 25% (1/4) stated that screening was “too difficult”. Of those who had worked 1–4 years, 57.1% (4/7) stated screening was “too difficult”. Of those who had worked more than 10 years 50% stated screening was “quite a bit” difficult. Of those who felt screening was “too difficult” (defined as having answered “a lot”, “somewhat”, or “quite a bit”) 61.5% (8/13) felt it was also too expensive to screen. Among those who did not feel screening was “too difficult” 42.9% (3/7) felt it was too expensive to screen. Additionally, 15.8% (3/19) of participants strongly felt their patients have more important issues than cervical cancer. The majority of participants felt that they need “a lot” of additional training in order to successfully perform screenings. All of those who had been working longest, more than 10 years, felt they needed additional training. Only 26.7% felt they were too busy to screen women. With regards to access to supplies needed to screen (defined as having answered, “a lot”, “somewhat”, or “quite a bit”) 61.1% (11/18) felt they did not have the supplies needed to screen and 40.0% (6/15) felt they did not have a lab necessary to screen. The majority of those without access to supplies also did not have access to the necessary laboratory resources. Of those participants who felt that screening was too expensive, 45.5% stated they did not have the necessary lab resources, 81.8% stated they had trouble with supplies. Of those who stated they did not feel they had adequate supplies or lab resources to perform visual inspections, 71.4% were willing to perform cervical cancer screening by visual screening.

#### 3.1.3. Practices

**Figure 1:**
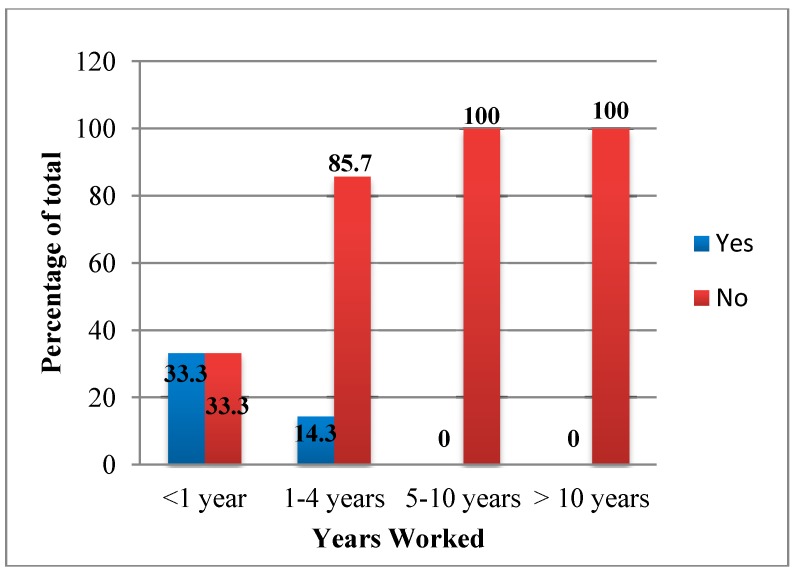
Percentage of respondents who have performed pap smears by amount of time worked.

Note: In those who have been working <1 year, the additional 33.3% is accounted for because one person stated they were unsure whether they had performed a pap smear.

With regards to current and past practice, when asked how long they had been working, 5/27 stated they had worked less than 1 year, 9/27 stated they had been working for 1–4 years, 4/27 stated they had worked 5–10 years, 3/27 stated they had worked longer than 10 years and 6/27 did not respond. Within the participants, 25% (6/24) stated they had performed cervical cancer screening of any kind. Only 16.7% (3/18) stated they had ever performed pap smears. Of those who had performed pap smears, only one stated they had performed more than 10 in their career. Figure 1 presents the percentage of respondents who have performed pap smears distributed by the length of time they have worked. Of those participants who stated they had been working for less than a year, 33% (1/3) stated they had performed a pap smear. Of the participants who stated they had been working more than 5 years, none of them had performed pap smears at all. No one stated they had performed visual inspection (with either acetic acid and Lugol’s solution).

### 3.2. Discussion

Our survey results indicated that there was a strong willingness within the healthcare workers of the Central Plateau to participate in a cervical cancer screening prevention campaign but they believe that screening is difficult and expensive. It also indicates that the health care workers feel that they need additional training, access to supplies, and a functional laboratory in order to perform the examinations. In addition, hardly any of the health care workers had performed cervical cancer screening and none had performed VIA. In terms of their knowledge base, only around half believed that cervical cancer is preventable, and a little less than half believed it was curable. However, the majority of participants believed that pre-cancerous cells could be detected. This discrepancy identifies a knowledge gap, most notably with the most recent data about the role of HPV in cervical cancer. A great number of the participants felt that cervical cancer could be identified early only through symptoms. With regard to knowledge of symptoms, a number of participants felt that a patient would have abdominal pain and/or loss of appetite in addition to abnormal bleeding. This shows that the health care workers are adept in recognizing more advanced symptoms of cervical cancer but may not recognize more subtle symptoms such as bleeding after intercourse or intermenstrual bleeding as concerning symptoms of early cancer. This is especially concerning because of the significant asymptomatic phase that accompanies cervical cancer that would surely be missed if this type of assessment were utilized. In addition, only a third of participants correctly answered there was a vaccine that could prevent cervical cancer. One reason for this significant knowledge gap could be the prohibitive cost of the HPV vaccine in a low-resource country. There was also a great discrepancy in the knowledge of risk factors between those who felt they were at risk for cervical cancer and those who felt they were not at risk. Those who felt they are at risk for cervical cancer correctly identified HPV infection and multiple partners as risk factors, whereas, those who did not admit to risk for self, identified poor hygiene and IUD use as risk factors for cervical cancer. The lack of recognition of risk factors is not only detrimental to the healthcare workers themselves but also to their patients that they are counseling. With that said, a large number of participants did recognize their own lack of knowledge and felt they needed additional training to feel comfortable with cervical cancer screening and prevention. Therefore, a great deal of education needs to be undertaken to help dispel myths and adequately train the healthcare workers. The highlighted knowledge gaps present a clear opportunity for education and training of healthcare workers about cervical cancer with provision of adequate resources.

With regards to their attitudes, all of our participants felt a cervical cancer-screening program should be started in their community. This is very encouraging considering the barriers they felt were limiting their ability to have a screening program in their community. Even the majority of those who stated they need additional education about cervical cancer were still willing to participate in a “screen and-treat” program. The barriers that were recognized by the health care workers were difficulty of performing screening, expense, and access to supplies and a functional laboratory. The benefit of VIA and the careHPV DNA tests is that both are very cost-effective and do not require any extensive supplies or laboratory functions. These are the best options for our resource-limited communities and the health care workers in the Central Plateau that were surveyed most definitely recognized themselves as resource limited. So in addition to adequate education and training, once shown how cost-effective and simple these techniques truly are for their resource-limited settings, we have an excellent opportunity to educate and train healthcare workers in the Central Plateau.

Lack of experience was recognized as a major barrier to providing cervical cancer prevention services. Only one out of the 27 participants had performed more than 10 pap smears and no one had performed visual screening. This suggests that the health care workers are not being trained on cervical cancer screening, which most likely has to do with the fact that pap smears are high-resource and high-expense screening practices. However, we did note that those who have been trained more recently were more likely to have performed cervical cancer screening although the number of respondents here was small. This is encouraging and may suggest a shift towards the training of healthcare workers in the technique of performing pap smears. Health care workers, as stated before, would need a great deal of additional training; fortunately there are standardized training efforts available in order to provide health care workers with the knowledge and confidence to successfully and efficiently perform visual screening and/or pap smears.

Cytology screening programs involving repeated screenings are highly effective at detecting precancerous lesions and preventing cervical cancer. The introduction of routine Papanicolaou (Pap) smears (and diagnostic biopsy coupled with treatment of precursor lesions) into high-resource European countries and North America was followed by a substantial reduction in disease burden. Despite the success of cytology-based screening programs, these programs rarely exist in low-resource regions and where they do exist are frequently ineffective, primarily due to inadequate technical, manpower and financial resources [10]. The multiple follow-up visits required following cytology-based screening is also a significant barrier to success in low-resource settings due to difficulties regarding contacting patients for follow up, as well as patient related issues of transportation, clinic hours, costs, child care needs and other factors. Studies suggest that cervical cancer screening programs are most successful and cost-effective in low-resource settings when they require few visits and offer a “screen and treat” for (single-visit) approach [10]. Visual inspection with acetic acid (VIA) [11] and a new rapid, low-cost simple version of HPV DNA testing (careHPV; QIAGEN, Gaithersburg, MD, USA) [12] are two cervical cancer screening alternatives to cytology that have been used in low and medium resource settings [12,13,14,15,16,17,18,19,20]. The VIA method involves applying a 3% to 5% solution of acetic acid (ordinary table vinegar) to the cervix followed by a naked eye assessment (no light source or magnification required) of the cervix a minute later [11]. Precancerous lesions and other abnormalities are apparent to the naked eye as areas of whiteness. VIA has been found to be low-cost, simple to perform, and can be incorporated into a single-visit approach [13,15,20].

In addition, the newly available careHPV test can be performed without electricity or running water in as little as 2.5 h [12] and therefore it may prove to be effective as part of a single-visit approach also. Cost-effectiveness analysis of cervical cancer screening approaches in less-developed countries have concluded that VIA as part of a well-organized “screen-and-treat” program can reduce cervical cancer mortality at low costs [21,22]. It demonstrated clinical effectiveness in over 1 million women in clinical studies conducted in China, Nigeria, Rwanda and Thailand. In terms of cost per life year saved, an analysis based on a rural Chinese setting found VIA to be more cost-effective than careHPV DNA testing, although VIA was not quite as effective at reducing cancer incidence [23]. In Nigeria, careHPV test was performed in challenging remote settings with minimal interventions and training from skilled professionals [24]. The Nigeria trial showed high specificity and adequate sensitivity with local investigators without any research background. This is very encouraging considering the knowledge and resource limitations of the Haitian healthcare workers.

**Figure 2:**
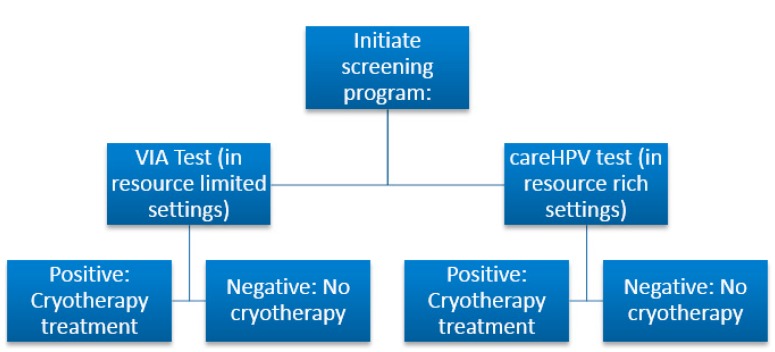
Screen-And-Treat Algorithm for Rural Haiti.

Note: Adapted from the WHO guidelines for Screening and Treatment of Precancerous Lesions for Cervical Cancer Prevention.

A single-visit approach combining VIA and cryotherapy when assessed through a demonstration project in rural Thailand was found to be safe, acceptable and feasible [16], and such an approach performed by nurses has also been found to be effective, safe and acceptable in India [20]. This is an excellent option for our population in Haiti. The cure rate for cryotherapy has been reported to be between 70%–88% depending on the setting and the progression of the precancerous lesions identified [20,25]. Due to VIA having a relatively high false positive rate in some settings (specificity reported to range from 64%–98% [13] there is a real possibility of over-treatment with cryotherapy; however, since cryotherapy is effective and safe, the overall risk-benefit-ratio is considered favorable. With this study and our study in mind, we feel most strongly that VIA and cryotherapy would be the most effective and cost-conscious “screen-and-treat” option for rural Haiti. Figure 2 illustrates a proposed algorithm for screen-and-treat programs that we feel would be most beneficial to rural Haiti.

A limitation to our study is our small sample size of participants. Due to the rural setting in which we performed this study there are fewer health care workers in the region to begin with, leading to a lower potential sample size overall. Many of these health care workers in the Central Plateau of Haiti take care of an entire neighborhood of individuals and work with other community members to disseminate information. Another limitation is missing information, whether due to lack of understanding of the question or inability to answer, a number of our questions had a large number of missing participants. In addition to missing information, each question had a variable number of participants answer them. This is likely due to the fact there were a significant number of incomplete surveys due to health care workers leaving questions blank. Due to such a significant number of incomplete surveys there are a number of questions for which the sample size is very low, which impacts our ability to make strong conclusions regarding the content of the question.

With the limitations in mind it is very important to remember that one of the greatest strengths of this study is that we are the first to perform a KAP study about cervical cancer screening in the Central Plateau of Haiti. Due to Haiti’s limited resources, cervical cancer is not being treated very often and therefore, the feeling prior to recent policy changes in the Haitian Ministry of Health, was that screening was not a priority for the patient population. Despite our small sample size, we feel that the qualitative information we elicited is extremely valuable. With improving access to treatment and the ever-growing presence of aid organizations such as Project Medishare and Partners in Health in the rural areas of Haiti, we have an opportunity to make a difference with a cost-efficient and simple method of “screen-and-treat”.

## 4. Conclusions

Our data establishes the fact that knowledge is fairly lacking even among healthcare workers and there is a clear opportunity to train healthcare workers in simple, cost effective “screen-and-treat” programs that could have a very great impact on the overall health of the population. Future plans include putting together a financial plan comparing visual screening and cryotherapy with HPV DNA testing and treatment to decide which is most cost effective and best suited for our specific population and setting. Then we plan to partner with local aid groups to provide training and supplies in order to pilot a “screen-and-treat” program in the Central Plateau.

## Supplementary Files

Supplementary File 1
