# Supplementary material for: Knowledge, Attitudes and Practices Regarding Cervical Cancer and Screening among Haitian Health Care Workers
Source: Int J Environ Res Public Health. 2014 Nov 10;11(11):11541–52. doi: 10.3390/ijerph111111541 (PMC4245628; doi:10.3390/ijerph111111541)
Supplement: Supplementary File 1 [file ijerph-11-11541-s001.pdf]

Knowledge, Attitudes and Practices Regarding Cervical Cancer and Screening among Haitian Health Care Workers

KAP Survey

Healthcare Provider Survey about Cervical Cancer

Thank you for taking the time to complete this survey about your experiences with screening for, diagnosing and treating cervical cancer. This is an anonymous survey. Neither your name (nor any other identifying information) will be collected or linked to your responses on this survey, so please respond to each question as accurately and honestly as possible.

First, we are interested in your thoughts about cervical cancer. Please indicate whether you believe each of the following statements is "True" or "False" by circling your answer.

	True (1)	False (2)	I Don't Know (3)
1. Cervical cancer is one of the leading causes of death in women worldwide.	1	2	3
2. Cervical cancer is preventable.	1	2	3
3. Cervical cancer is not curable.	1	2	3
4. It is possible to detect pre-cancerous cervical cells.	1	2	3
5. For cervical cancer, the progression of precancerous cells to cancer can take 10–20 years.	1	2	3
6. Cervical cancer is most common among women in their 20's.	1	2	3
7. Cervical cancer can usually be found at an early stage because of the obvious symptoms such as bleeding and pain.	1	2	3
8. If untreated, cervical cancer is fatal.	1	2	3
9. Cervical cancer is caused by a virus that is spread sexually.	1	2	3
10. There is a vaccine that can prevent cervical cancer.	1	2	3

11. Which of the following are risk factors for cervical cancer? (Check all that apply):

- Use of tampons
- Smoking cigarettes
- Infection with human immunodeficiency virus (HIV)
- Poor personal hygiene
- Infection with human papillomavirus (HPV)
- Use of intrauterine devices (IUDs)
- Having multiple sex partners
- Use of herbal remedies

12. Which of the following are symptoms of advanced cervical cancer? (Check all that apply):

- Abdominal pain
- Headaches
- Abnormal bleeding
- Foul-smelling discharge

- Nausea
- Loss of appetite

Next, we are interested in your thoughts about screening for cervical cancer. Please indicate whether you believe each of the following statements is "True" or "False" by circling your answer.

	True (1)	False (2)	I Don't Know (3)
13. The purpose of screening for cervical cancer is to detect pre-cancerous changes.	1	2	3
14. Screening for cervical cancer should ideally begin when a woman is in her twenties.	1	2	3
15. Screening for cervical cancer should ideally take place once a year.	1	2	3

16. Have you ever heard of any of the following tests? (**Check all that apply**):

- Pap smear
- Liquid-based cytology
- HPV DNA testing
- Visual inspection with acetic acid
- Visual inspection with Lugol's solution

17. The primary purpose of conducting a Pap smear is________________ (**Check only one**):

- To treat cervical cancer
- To cleanse the womb following sexual activity
- To detect pre-cancerous cervical lesions
- To ensure fertility
- To detect uterine cancer

For the following questions, please indicate the degree to which you agree or disagree with the below statements by circling your answer.

title	Strong Agree (1)	Agree (2)	No Opinion (3)	Disagree (4)	Strong Disagree (5)
18. Cervical cancer screening is an essential part of women's healthcare.	1	2	3	4	5
19. (For female respondents): I am not personally at risk for developing cervical cancer. (For male respondents): My girlfriend/wife is not at risk for developing cervical cancer.	1	2	3	4	5
20. Cervical cancer is a very serious disease.	1	2	3	4	5
21. I feel that I do not have adequate knowledge about cervical cancer.	1	2	3	4	5
22. Precancerous cervical cells are easy to detect.	1	2	3	4	5
23. Cervical cancer is difficult to treat.	1	2	3	4	5
24. A cervical cancer screening program should be started in my community.	1	2	3	4	5

Next, we are interested in some of your clinical experiences with cervical cancer screening, diagnosis and treatment.

25. Have you ever conducted cervical cancer screening? (**Check only one**):

- Yes
- No
- I don't remember/unsure

26. Have you ever performed a Pap smear? (**Check only one**):

- Yes (please answer the next question)
- No (please skip to question #29).
- I don't remember/unsure (please skip to question #29).

27. If you have performed a Pap smear, approximately how many have you *ever* performed? (**Check only one**):

- 1–5
- 6–10
- >10

28. How comfortable are you performing a Pap smear? (**Check only one**):

- Not at all comfortable
- Somewhat comfortable
- Quite comfortable
- Very comfortable

29. (For female respondents): Have you ever received a Pap smear? (**Check only one**):
(For male respondents): Has your wife or girlfriend ever received a Pap smear? (**Check only one**):

- Yes
- No
- I don't remember/unsure

30. Have you ever performed a visual inspection with acetic acid or Lugol's solution? (**Check only one**):

- Yes (please answer the next question)
- No (please skip to question #33).
- I don't remember/unsure (please skip to question #33).

31. If you have performed a visual inspection with acetic acid or Lugol's solution, approximately how many have you performed? (Check only one):

- 1–5
- 6–10
- >10

32. How comfortable are you performing a visual inspection with acetic acid or Lugol's solution? (Check only one):

- Not at all comfortable
- Somewhat comfortable
- Quite comfortable
- Very comfortable

33. Would you be willing to conduct cervical screening (using either a Pap smear or visual inspection with acetic acid or Lugol's solution) with future patients? (**Check only one**):

- Not likely
- Somewhat likely
- Very likely

We are interested in which factors make it harder to screen for cervical cancer. For the following factors, please indicate t*he extent to which each is a barrier* to screening for cervical cancer by circling your answer.

	Not at all (1)	Somewhat (2)	Quite a bit (3)	A lot (4)
34. My patients dislike/refuse screening.	1	2	3	4
35. My patients have more pressing health issues/problems.	1	2	3	4
36. I have not had the necessary training I need in order to screen.	1	2	3	4
37. The screening tests are too expensive for my patients.	1	2	3	4
38. I do not have enough time/I am too busy to screen.	1	2	3	4
39. I do not have the necessary supplies/equipment to screen.	1	2	3	4
40. The screening procedures are too difficult.	1	2	3	4
41. I do not (or my clinic/hospital does not) have the necessary laboratory resources to screen.	1	2	3	4
42. I do not (or my clinic/hospital does not) have the capacity to follow-up with patients following screening.	1	2	3	4

43. Have you ever diagnosed a patient with cervical cancer? (**Check only one**):

- Yes
- No
- I don't remember/unsure

44. Have you ever diagnosed pre-cancerous cervical lesions in a patient? (Check only one):

- Yes
- No
- I don't remember/unsure

45. Have you ever treated pre-cancerous cervical lesions in a patient? (Check only one):

- Yes (please answer the next question)
- No (please skip to question #47).
- I don't remember/unsure (please skip to question #47).

46. If you have treated pre-cancerous cervical lesions in a patient, what methods have you used? (**Check all that apply**):

- Hysterectomy
- Cone biopsy
- LEEP
- Cryotherapy
- Electro-diathermy
- Laser ablation

- I don't remember
- Other (specify): __________________

47. Have you ever treated invasive cervical cancer in a patient? (**Check only one**):

- Yes (please answer the next question).
- No (please skip to question #49).
- I don't remember/unsure (please skip to question #49).

48. If you have treated invasive cervical cancer in a patient, what methods have you used? (**Check all that apply**):

- Surgery
- Radiation
- Chemotherapy
- I don't remember
- Other (specify): __________________

Finally, we would like to know a little more about you. Please answer the below questions as honestly and accurately as possible.

49. How old are you (in years)? I am ________ years old.

50. What is your biological sex? (**Circle one**):

- Male
- Female

51. To which of the below ethnic groups to you belong? (Check all that apply):

- Haitian
- Marabou
- Taino
- Other (specify): __________________

52. What is your primary language? (Check only one):

- Creole
- French
- English
- Other (specify): ___________________

53. What is your primary job title? (**Check only one**):

- Obstetrics/gynecology faculty physician
- Obstetrics/gynecology community physician
- Non-obstetrics/gynecology faculty physician
- Non-obstetrics/gynecology community physician
- Resident physician
- Medical student
- Nurse
- Midwife
- Other (please specify): __________________________

54. How long have you worked under the current job title you selected above? (**Check only one**):

- Less than 1 year
- 1–4 years
- 5–10 years
- Longer than 10 years

You have completed the survey. We appreciate you taking the time to share your thoughts with us!

© 2014 by the authors; licensee MDPI, Basel, Switzerland. This article is an open access article distributed under the terms and conditions of the Creative Commons Attribution license (http://creativecommons.org/licenses/by/4.0/).
